# Conformational flexibility of adenine riboswitch aptamer in apo and bound states using NMR and an X-ray free electron laser

**DOI:** 10.1007/s10858-019-00278-w

**Published:** 2019-10-12

**Authors:** Jienv Ding, Monalisa Swain, Ping Yu, Jason R. Stagno, Yun-Xing Wang

**Affiliations:** Protein-Nucleic Acid Interaction Section, Structural Biophysics Laboratory, Center for Cancer Research, National Cancer Institute, National Institute of Health, Frederick, MD 21702 USA

**Keywords:** Adenine riboswitch, Aptamer, CPMG, Conformational exchange, B-factor

## Abstract

Riboswitches are structured *cis*-regulators mainly found in the untranslated regions of messenger RNA. The aptamer domain of a riboswitch serves as a sensor for its ligand, the binding of which triggers conformational changes that regulate the behavior of its expression platform. As a model system for understanding riboswitch structures and functions, the *add* adenine riboswitch has been studied extensively. However, there is a need for further investigation of the conformational dynamics of the aptamer in light of the recent real-time crystallographic study at room temperature (RT) using an X-ray free electron laser (XFEL) and femtosecond X-ray crystallography (SFX). Herein, we investigate the conformational motions of the *add* adenine riboswitch aptamer domain, in the presence or absence of adenine, using nuclear magnetic resonance relaxation measurements and analysis of RT atomic displacement factors (B-factors). In the absence of ligand, the P1 duplex undergoes a fast exchange where the overall molecule exhibits a motion at *k*_*ex*_ ~ 319 s^−1^, based on imino signals. In the presence of ligand, the P1 duplex adopts a highly ordered conformation, with *k*_*ex*_~ 83 s^−1^, similar to the global motion of the molecule, excluding the loops and binding pocket, at 84 s^−1^. The *µs*–*ms* motions in both the apo and bound states are consistent with RT B-factors. Reduced spatial atomic fluctuation, ~ 50%, in P1 upon ligand binding coincides with significantly attenuated temporal dynamic exchanges. The binding pocket is structured in the absence or presence of ligand, as evidenced by relatively low and similar RT B-factors. Therefore, despite the dramatic rearrangement of the binding pocket, those residues exhibit similar spatial thermal fluctuation before and after binding.

**Electronic supplementary material:**

The online version of this article (10.1007/s10858-019-00278-w) contains supplementary material, which is available to authorized users.

## Introduction

Riboswitches are structured RNAs that selectively bind metabolites for controlled gene expression. A riboswitch consists of two domains: an “aptamer” and an “expression platform” (Mandal and Breaker 2004; Winkler and Breaker 2003). Binding of a metabolite to the aptamer domain leads to an allosteric conformational change that is transmitted to the expression platform via a structurally regulated “switching” sequence (Batey 2012), enabling control over gene expression (Peselis et al. 2015; Wickiser et al. 2005). Purine riboswitches, the largest known class of riboswitches, include transcriptional regulators of *pbuE* (on-switch) and *xpt*-*pbuX* (off-switch), and the translational regulator of *add* (on-switch). Although the mode of gene regulation by riboswitches is diverse, the ligand recognition by their aptamer domains is relatively conserved across classes (Mandal and Breaker 2004; Winkler and Breaker 2003). The structural dynamics of the *add* (adenosine deaminase) adenine (A) riboswitch has been studied (Warhaut et al. 2017; Lee et al. 2010; Reining et al. 2013). Previous studies of the adenine-sensing aptamer domain revealed that the ligand-free state is conformationally heterogeneous, but in the presence of Mg^+2^ is pre-organized for adenine binding by forming kissing-loop interactions (Lee et al. 2010; Noeske et al. 2007; Nozinovic et al. 2014; Reining et al. 2013). In other studies, however, the ligand-binding pocket was suggested to be locally disordered in the absence of ligand (Batey 2012; Gilbert et al. 2006), and that rearrangement and stabilization of the pocket occur upon ligand binding (Gilbert et al. 2006; Di Palma et al. 2013). Our recent real-time, ligand-triggered crystallography study using XFEL determined structures of two apo states (apo1/apo2), a transient intermediate ligand-bound state (IB·ade), and the final bound state formed *in crystallo* (B·ade) (Stagno et al. 2017). The presence of four conformational states agreed with the four-state kinetic model in solution, thus revealing the structural basis for the switching action (Stagno et al. 2017). The crystallographic data, i.e. B-factor, which was recorded at room temperature, may provide a unique insight into a spatial aspect of molecular motion and complement the information from the *µs*-*ms* conformational dynamics to present a complete view of the RNA motion. Here we present the conformational flexibility of the aptamer domain in both the ligand-free and ligand-bound states using solution NMR spectroscopy and RT B-factor analysis derived from the crystallographic experiments using XFEL. We used spin relaxation and relaxation compensated Car-Purcell-Meiboom-Gill (rc-CPMG) dispersion experiments to observe the signals in hetero-nuclear single quantum coherence (HSQC) spectra of imino groups. Combined spatial and temporal information provides atomic-level details and timescales associated with the binding and switching process, thereby furthering our understanding of the regulatory mechanism of purine-riboswitches.

## Materials and methods

### RNA sample preparation

The two strands of the DNA template, for transcription of the 71-nucleotide (nt) aptamer domain of the *add* A-riboswitch (rA71), were synthesized by Integrated DNA Technologies (IDT). The sequence of the template strand is as follows:

5′-TCT GAT TCA GCT AGT CCA TAA TAC GAC TCA CTA TAG GGA ACA TAT AAT CCT AAT GAT ATG GTT TGG GAG TTT CTA CCA AGA GCC TTA AAC TCT TGA TTA TGT TCC C-3′

The template strand contains an 18-nt spacer sequence prior to the T7 promoter (shown as underlined). This spacer gives flexibility to the template strand and helps to reduce molecular crowding when binding to the agarose beads. It also reduces steric hindrance for binding of the T7 RNA polymerase and increases the binding efficiency. First, the two complementary DNA strands were dissolved in water and annealed at room temperature. The double-stranded DNA was then used as template for touch-down PCR (TD-PCR) (Liu et al. 2015).

Primer sequences used for TD-PCR are as follows:

Forward primer: 5′-/Bio/TCT GAT TCA GCT AGT CCA TAA TAC GAC TCA CTA TAG G-3′

Reverse primer: 5′-mGmGG AAC ATA ATC AAG AGT TT-3′

The forward primer is 5′ biotinylated and the reverse primer contains two 2′-O-methyl guanosines (mG) at the 5′ end, which reduce the non-templated nucleotide addition to the transcription product. Both primer sequences were purchased from IDT.

The TD-PCR reaction mixture contained the following:

0.001 μM double stranded DNA template

0.001 mM primers

0.2 mM NTPs

Taq DNA polymerase reaction buffer (10 mM Tris-HCl, 50 mM KCl, 2 mM MgCl_2_, pH 8.0).

A TD-PCR was performed as reported previously (Liu et al. 2015) with some modifications. The first step: 95 °C for 2 min, followed by the touch-down phase and PCR phase. The touch-down phase starts with 95 °C for 30 s, annealing for 45 s followed by elongation at 72 °C for 40 s. The annealing step of the touch-down phase has a temperature ramp from 75 to 45 °C in 20 cycles (1.5 °C per cycle). The PCR phase has 45 thermal cycles, and each cycle has melting at 95 °C for 30 s, annealing at 50 °C for 45 s, and elongation at 72 °C for 40 s.

### Template attachment and transcription

The template preparation and transcription were performed according to the method described previously (Liu et al. 2015). Briefly, commercially purchased neutravidin (Thermo Fisher Scientific) coated agarose beads (30–165 μm diameter) were used as solid-phase support. First, the neutravidin beads were washed with water, then 3 times with buffer A (10 mM Tris-HCl, 50 mM KCl, pH 8.0). The PCR product (10 ml) was incubated with neutravidin beads at room temperature overnight to immobilize the DNA template. The next day, the beads were added to a Pierce Centrifuge column (~ 30 μm pore size) and centrifuged at 500 rpm, 4 °C for 1 min. The beads were then washed 3 times with buffer A. Approximately 80% of the template could be attached to the neutravidin beads. The bead-attached templates were stable for weeks and reusable for multiple RNA preparations. In vitro transcription was used to prepare all RNA samples, which includes the following steps for a 10 ml transcription reaction. DNA templates attached neutravidin agarose beads were incubated with 80 mM HEPES-KOH (pH 7.5), 28 mM MgCl_2_, 2 mM Spermidine, 40 mM DTT, 6 mM rNTPs, T7, SUPERase In RNase Inhibitor (Invitrogen) and deionized H_2_O to a final volume of 10 ml for 3 to 5 h. The RNA product was purified by urea-denaturing polyacrylamide gel electrophoresis, eluted from gel by RNA elution buffer (0.3 M sodium acetate, 2 mM EDTA, pH 5.3) at 4 °C overnight and finally buffer changed to NMR buffer (10 mM potassium phosphate, 30 mM KCl, 2 mM MgCl_2_, pH 6.8).

### NMR sample preparation

^15^N uniformly labeled rA71 samples were made by the above in vitro T7 transcription protocol using ^15^N-labeled rNTPs. The final RNA concentration used for NMR measurements was 0.6 mM. For bound rA71, adenine was added to a final concentration of 5 mM.

### NMR relaxation experiments

#### NMR spectroscopy

NMR relaxation experiments were performed on Bruker Avance spectrometers operating at proton frequencies of 850 MHz, 700 MHz or 600 MHz. 2D ^1^H-^15^N TROSY-HSQC and 3D ^15^N NOESY-HSQC spectra were recorded on the 850 MHz spectrometer. All spectrometers are equipped with proton-cooled cryogenic ^1^H/^13^C/^15^N triple resonance probes. Sample temperature was calibrated with a 100% methanol sample prior to each experiment.

#### *R*_*1*_, *R*_*2*_ and [^1^H]-^15^N NOE experiments

The *R*_1_, *R*_2_ and [^1^H]-^15^N NOE experiments were performed at 25 °C on the 700 MHz spectrometer. These data were acquired by implementing band selective excitation pulses for imino protons, and are mainly based on the standard pulse sequences by Farrow et al. (1994). For *R*_1_ and *R*_2_ experiments, a recycle delay of 2.5 s was used. *R*_1_ values were measured by a pseudo-3D experiment by looping the *R*_1_ delays of 100(× 2), 200, 400, 700, 1000, 1300, 1700, 2100 ms. *R*_2_ measurements were obtained using on-resonance rotating frame relaxation experiments. A series of *R*_1_ experiments were collected under 1.5 kHz spin-lock field with different spin-lock durations: 1, 10(× 2), 20, 40, 60, 100, 160 and 220 ms. For the [^1^H]-^15^N NOE experiments, one was collected without saturation, and the other with proton saturation for 3.0 s. The *R*_1_ and *R*_2_ values were obtained by fitting the peak heights to a single exponential function using a nonlinear least squares method. The *R*_2_ values were calculated based on the definition (Palmer and Massi 2006):

1 $$R_{2} = R_{1\rho } /\sin^{2} \theta - R_{1} \tan^{2} \theta$$

where the tilt angle in the rotating frame is defined as:

2 $$\tan \theta = \frac{2\pi \Delta \nu }{{\gamma_{N} B_{1} }}$$

where ∆ is the resonance offset and $\frac{{\gamma_{N} B_{1} }}{2\pi }$ is the strength of the spin-lock field.

The imino [^1^H]-^15^N NOE values were obtained from the ratio of peak intensities in the saturated spectrum to those in the unsaturated spectrum. The error was estimated by calculating the standard deviations as follows:

3 $$\sigma_{NOE} /NOE = \left[ {\left( {\sigma I_{sat} /I_{sat} } \right)^{2} + \left( {\sigma I_{unsat} /I_{unsat} } \right)^{2} } \right]^{1/2}$$

## ^15^N CPMG relaxation dispersion experiments

The imino ^15^N TROSY-CPMG experiments were carried out at 25 °C for both apo and bound rA71. The imino ^15^N TROSY-CPMG data were collected on 600 MHz and 850 MHz spectrometers for 9 different $\nu_{cpmg}$ values of 0, 80, 160, 320, 480, 640 (× 2), and 800 Hz with a constant transverse relaxation time of 50 ms, and 66.67, 133.33 Hz with a constant transverse relaxation time of 60 ms. The NMR relaxation data were processed using NMRPipe (Delaglio et al. 1995) and analyzed using NMRView (Johnson 2004). Relaxation rates $R_{2}^{eff}$ were determined from peak heights using equation $R_{2}^{eff} = \left( {{{ - 1} \mathord{\left/ {\vphantom {{ - 1} T}} \right. \kern-0pt} T}} \right)\ln \left( {{{I_{{\nu_{cpmg} }} } \mathord{\left/ {\vphantom {{I_{{\nu_{cpmg} }} } {I_{0} }}} \right. \kern-0pt} {I_{0} }}} \right)$, where T is the constant transverse relaxation time, $I_{0}$ is the intensity in the reference spectrum, and $I_{{\nu_{cpmg} }}$ is the intensity at the different CPMG field strengths. Errors for $R_{2}^{eff}$ were estimated from the peak intensity differences ∆*I* of repeated experiments at $\nu_{cpmg}$ = 640 Hz, using $\sigma_{{\nu_{cpmg} }} = \Delta I/\left( {T \times I_{{\nu_{cpmg} }} } \right)$ equation. The results are reported only for those resonances that are not overlapped and that have sufficient signal to noise ratio. The quantitative information was extracted by fitting the transverse relaxation rates with equation:

4 $$R_{2} \left( {{1 \mathord{\left/ {\vphantom {1 {\tau_{cp} }}} \right. \kern-0pt} {\tau_{cp} }}} \right) = R_{2}^{0} + R_{ex} \left[ {{{1 - 2\tanh \left( {k_{ex} \tau_{cp} /2} \right)} \mathord{\left/ {\vphantom {{1 - 2\tanh \left( {k_{ex} \tau_{cp} /2} \right)} {k_{ex} \tau_{cp} }}} \right. \kern-0pt} {k_{ex} \tau_{cp} }}} \right]$$

where $R_{ex} = p_{a} p_{b} \Delta \omega^{2} /k_{ex}$. The equilibrium populations are given by *p*_*a*_(*p*_*b*_), *k*_*ex*_ is the sum of the forward and reverse rate constants for a two-site conformational equilibrium and Δω is the ^15^N chemical shift difference between the two conformations, *a* and *b*. The fitting was done using GLOVE software package (Sugase et al. 2013).

### Small angle X-ray scattering

The detailed procedure for SAXS data collection, processing and analysis are previously described (Wang et al. 2009) using an in-house program package NCI-SAXS or a program package by Svergun et al. (http://www.embl-hamburg.de/biosaxs/). The experimental radius of gyration (*R*_g_) was calculated from the data at low *q* values in the range of *qR*_g_ < 1.3, using the Guinier approximation of ln*I*(*q*) ≈ ln(*I*(0)) − *R*_g_^2^*q*^2^/3.

### B-factor analysis

The detailed description of serial femtosecond crystallography experiments using an XFEL, including the preparation of nano/microcrystals of rA71, data collection, data processing, and structure determination, were reported previously (Stagno et al. 2017). Briefly, using a “mix-and-inject” approach, adenine ligand (20 mM) was diffused into the flowing slurry of crystals, upstream of the X-ray interaction region. The delay time between ligand mixing and X-ray exposure was controlled by the path-length of HPLC tubing. Data were recorded in real-time at room temperature, and processed with Cheetah (Barty et al. 2014) and CrystFEL (White et al. 2012). The binning of data at a 10 s delay interval post-mixing revealed an intermediate bound (IB·ade) conformation. For B-factor analysis, the average B-factors per residue (Fig. 6) were extracted from the PDB coordinate files 5E54 (apo1/apo2) and 5SWE (B·ade) using *Baverage* from CCP4 suite (Winn et al. 2011). The spatial conformational flexibility of the four states was calculated based on the B-factors using PHENIX (Adams et al. 2002).

## Results and discussion

### NMR assignments for rA71 in absence or presence of ligand

**Fig. 1:**
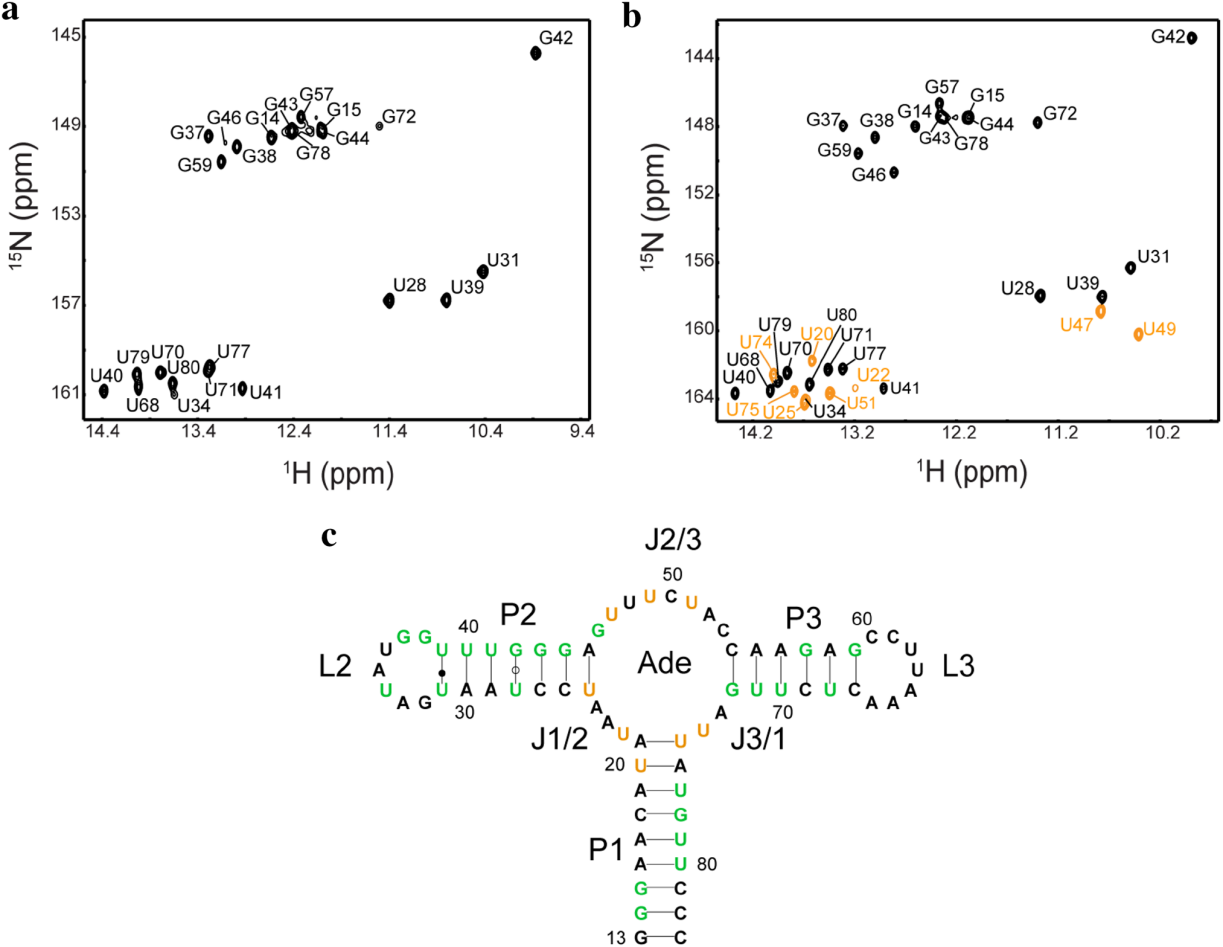
NMR assignments for rA71 in the absence or presence of adenine. **a**, **b** 2D ^1^H-^15^N HSQC spectra, with assignments, for rA71 in the apo (**a**) and bound (**b**) states. **c** Secondary structure of rA71. Residues assigned in both apo and bound states are colored in green, and those assigned only in the bound state are colored in orange

The imino assignments for the A-riboswitch aptamer domain in apo and bound states are shown in Fig. 1. The residues with assigned imino signals are mostly located in P1, P2, and P3 duplexes. The imino signals from all other residues were not detectable due to fast exchange with solvent (Allner et al. 2013; Noeske et al. 2007; Di Palma et al. 2015).

**Fig. 2:**
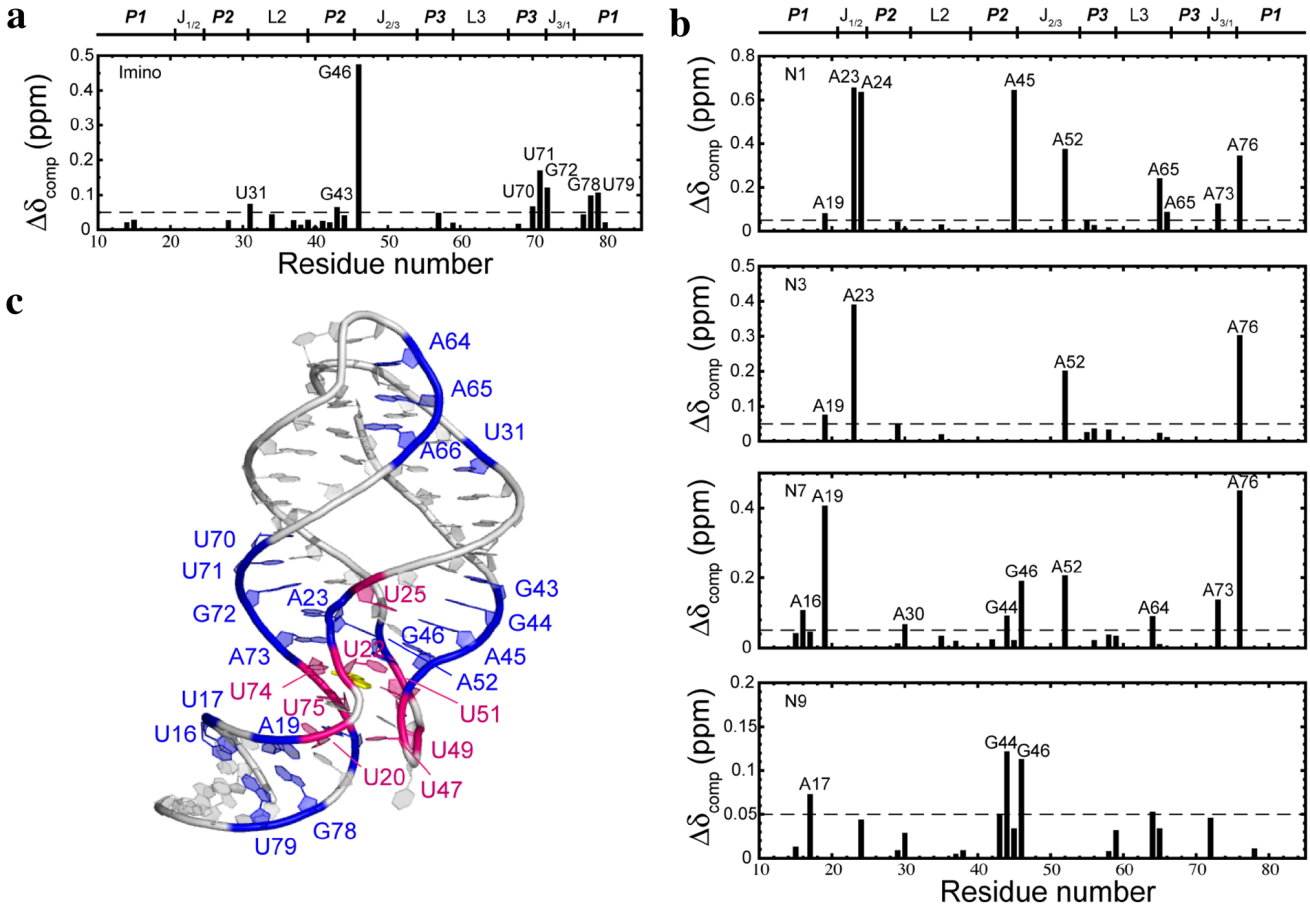
Composite chemical shift changes in imino (**a**) and N1, N3, N7, N9 (**b**) upon ligand binding, calculated using the empirical equation: $\Delta \delta_{comp} = \sqrt {\Delta \delta_{H}^{2} + \left( {\Delta \delta_{N} /6.5} \right)^{2} }$. The residues with chemical shift larger than 0.05 ppm (dotted line) are labeled. The regions of rA71 to which the residue numbers correspond are denoted on the top of the graph. **c** Residues of the signals that appeared (magenta) or with large chemical shift (blue) after ligand binding are highlighted in the crystal structure (PDB 1Y26)

In the presence of adenine, eight additional peaks, U20, U22, U25, U47, U49, U51, U74, and U75 (shown in orange in Fig. 1c), were detected and assigned, all of which, except for U20, reside in the binding pocket. U22, U47, U49, U51, and U74 are directly involved in ligand binding, consistent with previously reported crystal structures and MD simulations (Sharma et al. 2009; Gong et al. 2011; Priyakumar and MacKerell 2010). U20 and U75 form three base-triples U49·(U20-A76) and C50·(U75-A21), respectively, which are not observed in the apo state (Stagno et al. 2017; Serganov et al. 2004). U25 forms a Watson-Crick base pair with A45. In addition to the above residues, U31, G43 (P2); U70, U71, and G72 (P3); G78 and U79 (P1); and G46 (J2/3) exhibit significant chemical shift changes in response to adenine binding (Fig. 2a). Among those residues, only G46 is located in the binding pocket.

To obtain more information for the junction and loop regions where no imino signals were detected, we used long-range (N1, N3, N7 & N9) ^1^H-^12^C-^15^N two-bond coupling HSQC experiments. The ligand-induced chemical shift changes for these spins are shown in Fig. 2b. Besides the residues already detected by imino HSQC experiments, additional residues in P1 (A16, A17, A19 and A76), the binding pocket (A23, A24, G46, A52 and A73), P2 (G44 and A45), and L3 (A65 and A66), showed large chemical shift changes.

**Fig. 3:**
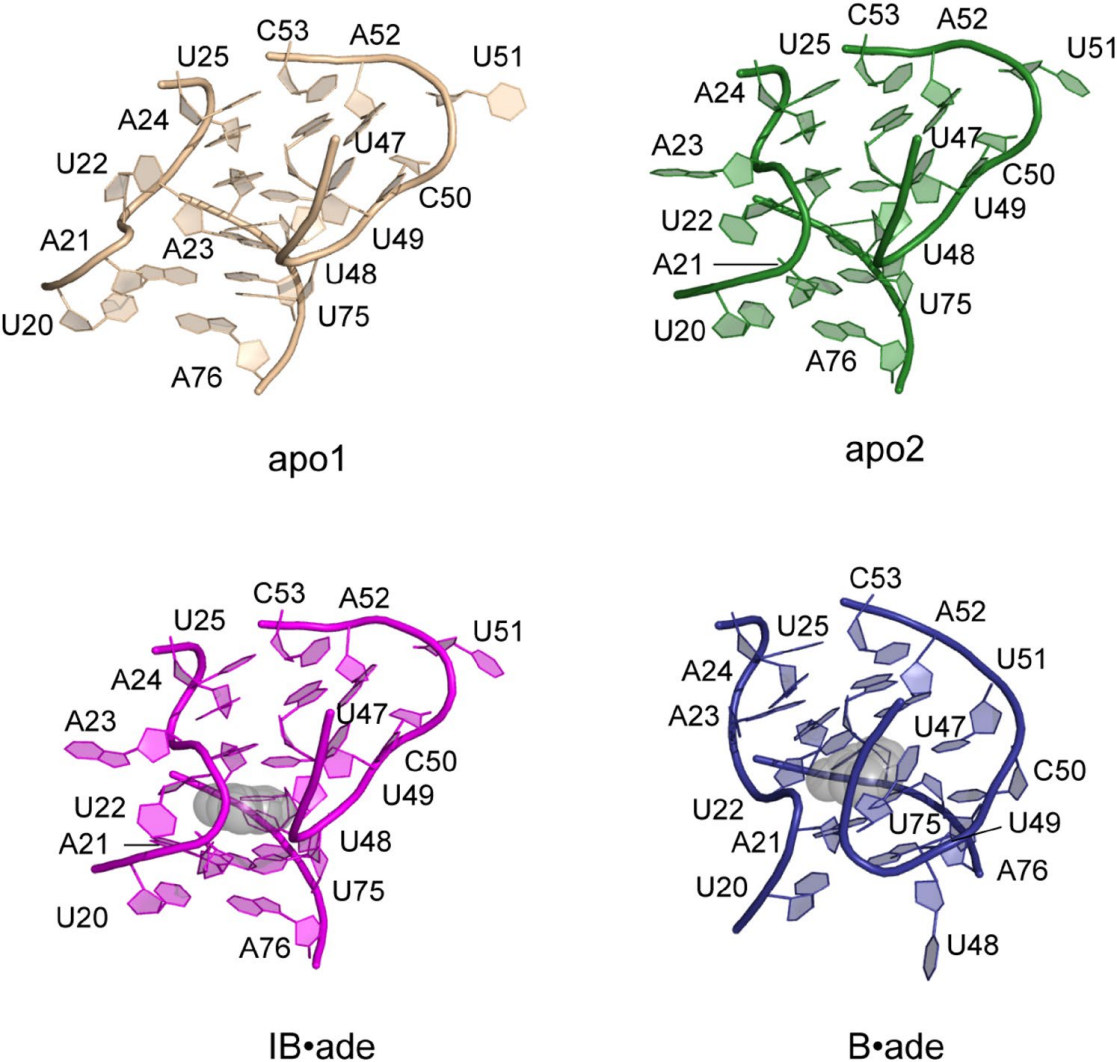
Structures of the binding pockets in each of the four conformational states: apo1, apo2, IB·ade, and B·ade. The binding pockets are structured in all four states, albeit with different sets of hydrogen-bonding and base-stacking interactions. The binding pocket structures of apo2 and IB·ade are closely related, with the displacement of U48 and A21 by the ligand as the only major difference

The assignments, together with chemical shift changes upon binding, give the general view of ligand-induced changes at the residue level. The greater number of assigned residues in the binding pocket in the presence of ligand indicates that solvent exchanges of those imino groups decreased due to either the direct binding of adenine or adenine-induced conformational changes. The chemical shift changes upon binding indicate structural rearrangements, and are consistent with crystal structure observations (Fig. 3). The P3-L3 hairpin and P1 seem to be more sensitive to ligand binding and undergo conformation changes of larger magnitude than the P2-L2 hairpin (Fig. 2c). This finding further supports the notion derived from our XFEL experiments that the ligand-induced changes to P1 are stabilized by aligning of the P1 and P3.

### Pico- to nano-second timescale conformational flexibility of rA71

**Fig. 4:**
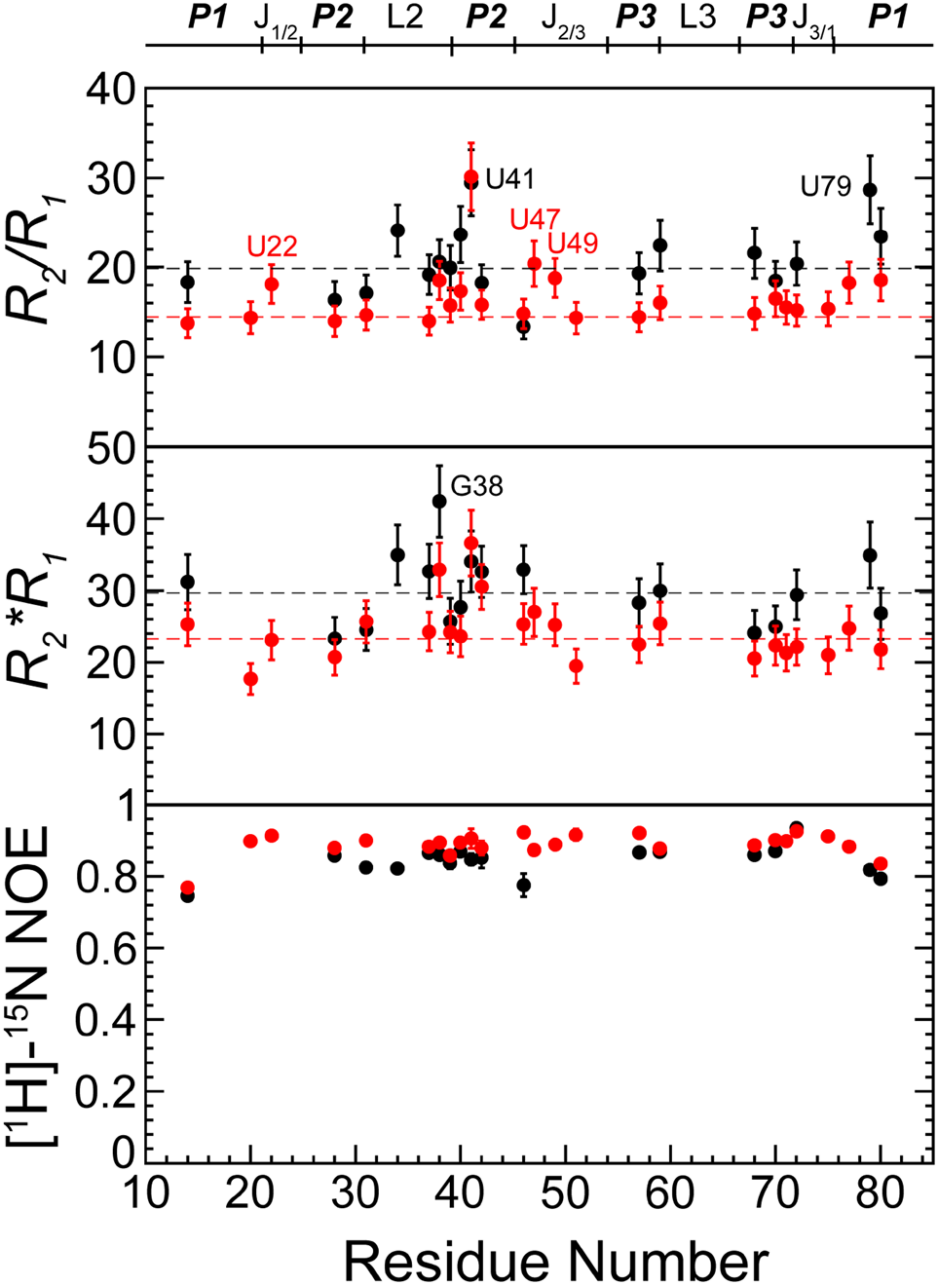
Imino ^15^N spin-relaxation measurements for rA71 in apo (black) and bound (red) states. The plots of *R*_2_*/R*_1_ (top), *R*_2_*R_1_ (middle), and [^1^H]-^15^N NOE (bottom) illustrate the pico- to nano-second timescale conformational flexibility of rA71. The average *R*_2_*/R*_1_, *R*_2_*R_1_ values for both apo and bound states are shown by dashed lines. Residues with higher values than the average in *R*_2_*/R*_1_ are labeled

We measured the ps-ns timescale backbone dynamics (*R*_1_, *R*_2_ and [^1^H]-^15^N NOE) of rA71 by monitoring the imino signals. Figure 4 shows the spin relaxation measurements for both apo and bound forms. *R*_2_*/R*_1_ and *R*_2_*R_1_ provide semi-quantitative insight into the dynamic properties, where *R*_2_*/R*_1_ values are well suited to evaluate the overall tumbling times of macromolecules undergoing diverse local mobility, while *R*_2_*R_1_ describes the amplitude of those local motions. In all three data sets (*R*_2_*/R*_1_, *R*_2_*R_1_, [^1^H]-^15^N NOE), the aptamer has distinctly different dynamics in the apo and bound states. We initially examined the global conformational flexibility by the average *R*_2_*/R*_1_ values. The residues with *R*_2_*/R*_1_ values greater than 1.5 standard deviations were excluded. The final values for rA71 in the absence or presence of ligand are 19.9 ± 1.4 and 14.4 ± 0.3, respectively. A significant decrease in *R*_2_*/R*_1_ for the bound state suggests an overall more compact structure. This result is consistent with our small angle X-ray scattering data where the gyration radius of rA71 in absence of ligand is 23.8 ± 0.3 Å, while in presence of ligand is 22.7 ± 0.3 Å. Residues with elevated *R*_2_*/R*_1_ values in the absence of ligand are mainly in the stem regions of P1 (U79, U80), P2 (U40, U41), and P3 (G59, U68), as well as in L2 (U34, G38). In the presence of ligand, residues with values slightly above the average *R*_2_*/R*_1_ are U77, U80 (P1); U39, U40, U41, G42 (P2); G59, U70 (P3); G38 (L2); U22 (J1/2); U47, U49 (J2/3). The additional regions showing conformational exchange in the bound state are in the binding pocket, where U22, U47 and U49 form three base-triples, A73·(A52-U22), U49·(U20-A76) and U51·(adenine-U74)·U47, that lock P1 and J2/3 junction together (Serganov et al. 2004). Furthermore, U41, which is located in the P2 duplex, shows elevated *R*_2_*/R*_1_ compared to the average in both the absence and presence of ligand.

The P1 stem, which harbors the “switching sequence” critical for *add* regulation, is dynamic in the apo state, indicated by both the increased *R*_2_*/R*_1_ and decreased [^1^H]-^15^N NOE values (Fig. 4). The dynamic behaviors among these residues have a poor correlation to one another, reflected by the random fluctuation in *R*_2_*/R*_1_ values. Such high flexibility might be caused by the opening of the P1 helix, as revealed by molecular dynamics simulations (Di Palma et al. 2013). In contrast, the fluctuation in the P1 stem is significantly reduced upon ligand binding, as suggested by the *R*_2_*/R*_1_ values closer to the average, consistent with the formation of a stable P1 duplex upon ligand binding. Stabilization of the P1 duplex upon ligand binding that results in regulation of gene expression downstream is a common feature among this class of riboswitches (Montange and Batey 2006; Huang et al. 2011; Haller et al. 2013; Suresh et al. 2016). With respect to the binding pocket, residues U49 and U51, which were not detected in the apo state, were stabilized upon binding, as reflected by their small *R*_2_*/R*_1_ values. Furthermore, the high [^1^H]-^15^N NOE values (~ 0.9) for the residues in this region, indicate that the imino groups of the local structure are ordered after adenine binding.

### Micro- to milli-second timescale conformational dynamics of rA71

Our previous kinetics study showed that the conformational exchanges among the four states are on subsecond-second timescales (Stagno et al. 2017). Nevertheless, as mentioned in the previous section, several residues in rA71 have elevated *R*_2_*/R*_1_ values in both apo and bound states, indicating possible micro- to-milli-second timescale conformational dynamics before and after binding. We then examined the relaxation dispersion data of rA71 in the absence or presence of ligand.

**Fig. 5:**
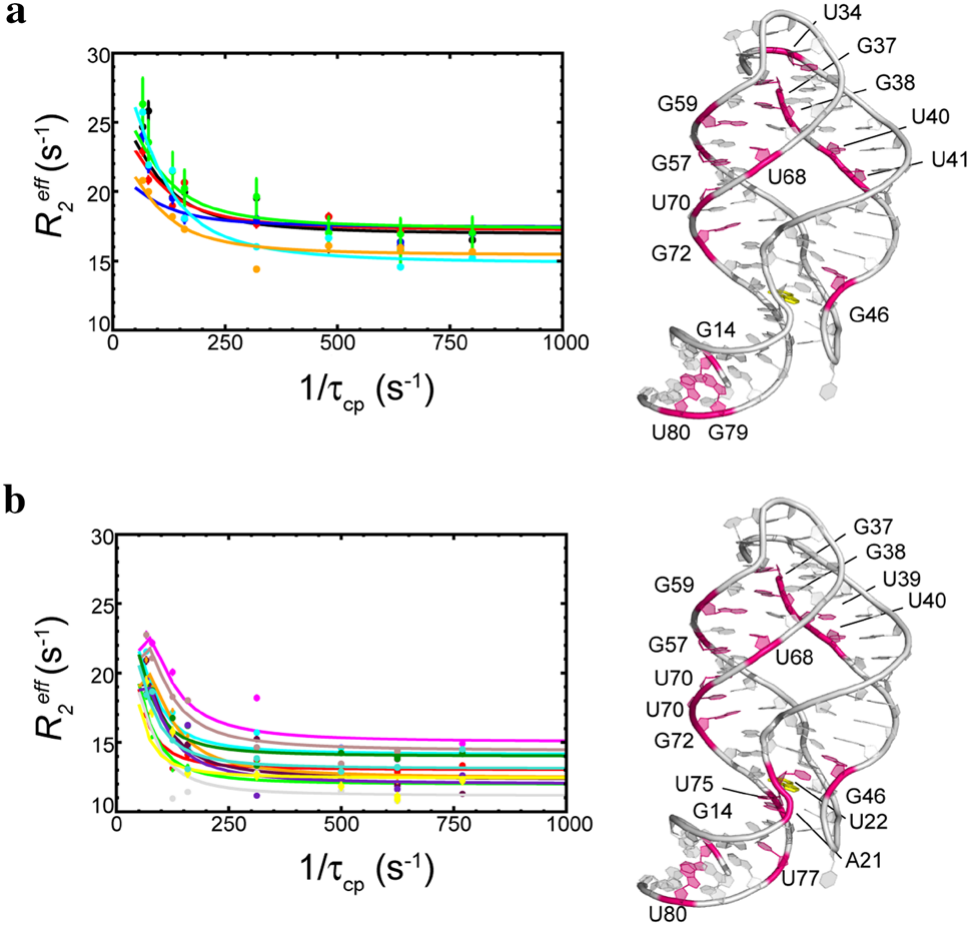
Micro- to milli-second timescale conformational exchanges of rA71 in apo (**a**) and bound (**b**) states by imino ^15^N CPMG relaxation dispersion experiments. The residues showing conformational exchanges identified by imino ^15^N CPMG relaxation dispersion experiments are mapped onto the structure (magenta). The relaxation dispersion profiles of representative residues are shown in the left panel using the data from 600 MHz. The fitting results are summarized in Tables S1, S2 and Fig. S3

In the absence of ligand, the imino CPMG results identified 14 residues (excluding residues with overlapping signals) with conformational flexibility on the *µs*-*ms* time scale (Table S1). Most of these residues are located in the stem regions of P1 (G14, U79, U80), P2 (U40, U41) and P3 (G57, G59, U68, U70, G72) (Fig. 5a). Very few signals from residues in L2 (U34, G37, G38) and J2/3 (G46) were observed, likely due to fast exchange with solvent. Among those, only 9 residues can be fit individually with reasonable errors (Table S1). Residues U41 and U79 have poor data quality, so their data are not used for the segmental or global fitting. While U80 in P1 could not be fit individually, the segmental fit using detected residues (G14, U80) in P1 yields *k*_*ex*_ = 3184 ± 32 s^−1^, which is less reliable due to the limited number of data points. *k*_*ex*_ of the residues from the rest of the structure based on the individual fit ranges from 137 to 448 s^−1^ and the global fit yields *k*_*ex*_ = 319 ± 1.4 s^−1^ (Table S2).

In the presence of ligand, a total of 17 resolved resonances show conformational exchange. Similar to the apo state, these sites are mainly located in helical regions: P1 (G14, U20, U75, U77, U80), P2 (U39, U40), and P3 (G57, U68, U70, U71, G72); few are sparsely distributed in loops and junctions: J1/2 (U22), J2/3 (G46) and L2 (G37, G38) (Fig. 5b). Individual fits yield *k*_*ex*_ ranging from 74 s^−1^ (G72) to 1194 s^−1^ (G59). The segmental fits for three duplexes, P1, P2, and P3, result in *k*_*ex*_ of 83, 94 and 89 s^−1^, respectively (Table S2), significantly lower than those in the absence of ligand (Table S2). It is interesting to note that *k*_*ex*_ for the binding pocket remains similar in the absence and presence of ligand (Table S1). But the lack of sufficient detectable signals from the residues in the binding pocket makes the fitting less reliable.

### B-factor analysis

**Fig. 6:**
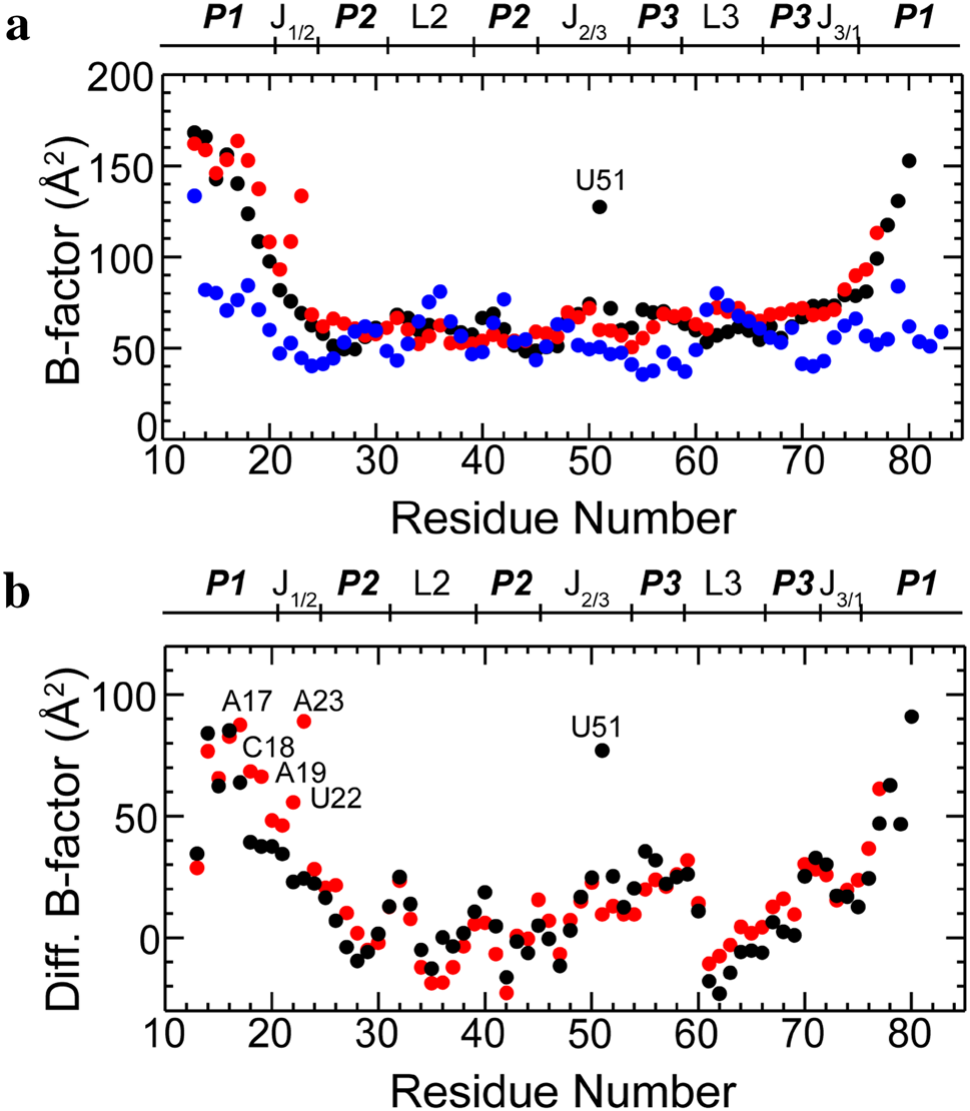
Comparison of RT B-factors for the apo and ligand-bound structures of rA71: apo1/apo2 (PDB 5E54), B·ade (PDB 5SWE). The residues with significant differences are labeled. **a** Plot of average B-factors per residue as reported in the crystal structures of apo1 (black), apo2 (red), and B·ade (blue) (Stagno et al. 2017). **b** Differences in the average B-factors per residue between apo1 and B·ade (black) and apo2 and B·ade (red). The outlier (U51) of apo1 has a large elevated B-factor, as it is completely solvent-exposed without any crystal contact with other RNA atoms in the crystal structure, whereas U51 of apo2 is stabilized by a crystal packing interface as well as a coordinated Mg^2+^ ion. In the bound conformation, U51 interacts directly with the adenine ligand

The relaxation dynamics alone do not provide direct spatial information with regard to atomic motion. For this, we analyzed the RT B-factors from the XFEL crystal structures (Fig. 6). The RT B-factors are associated with the spatial extent of thermal fluctuation and atomic static disorder (Drenth 1994) in a crystal structure. It has been illustrated that RT X-ray crystallographic data correlates with NMR relaxation measurements and that the combined use of these data provides a more complete spatial and temporal depiction of motion (Fenwick et al. 2014; Fulle and Gohlke 2008). For RNA, RT B-factors become particularly informative regarding the flexibility and static disorder of non-duplexed regions, where NMR imino signals may be undetectable. RT B-factors, together with three-dimensional structures, provide spatial information that complements temporal information provided by NMR relaxation measurements. Of note, diffraction data should be recorded at the same temperature as the NMR experiments, as B-factors are highly temperature-dependent (Fig. S2).

In general, the values of the RT B-factors are consistent with *k*_*ex*_ values from the global and segmental fittings (Table S2). The average RT B-factors for the two apo conformations (~ 77 Å^2^) (Fig. 6a; Table S2) is considerably higher than that observed for the bound conformation (~ 58 Å^2^), despite the fact that the apo crystal data are of better quality, and to higher resolution (2.3 Å for apo1/apo2 vs. 3.0 Å for B·ade). In particular, the P1 helix exhibits the greatest changes in B-factors (Fig. 6a), with average values of ~ 123, 131, and 69 Å^2^ for apo1, apo2 and B·ade, respectively. This reflects a high degree of atomic motion of residues in the P1 stem, most probably associated with duplex opening in the absence of ligand and stabilization of the duplex upon ligand binding. The dramatic changes in the RT B-factors of the P1 duplex upon ligand binding are clearly illustrated by plotting the differences of the RT B-factors between the apo and bound structures (Fig. 6b). We then performed ensemble calculation based on the B-factors of all four states using PHENIX (Adams et al. 2002) and results provide visualizations of comparison of spatial motions of the four states (Supplemental Video 1). The binding pocket, on the other hand, shows relatively small B-factor differences, despite large conformational changes (Fig. 3), which appears to be consistent with the *μs*-*ms* relaxation date (Table S2). This implies that the binding pocket, although temporally flexible, exhibits comparable spatial fluctuation before and after ligand binding.

## Conclusion

Our NMR relaxation data, combined with the RT XFEL structural information, provide temporal and spatial evidence for the differential conformational dynamics of the *add* riboswitch aptamer domain. Ligand binding reduces overall motions in all duplex regions, in particular, the P1 duplex based on both the relaxation data and the RT B-factors, and result in the more compact structure as indicated by the radius of gyration.

Previous kinetic data from stopped-flow experiments revealed a four-state model (Stagno et al. 2017).

5 $${\text{apo1}}\underset{{{\text{k}}_{cl} }}{\overset{{{\text{k}}_{op} }}{\rightleftharpoons}}{\text{apo}}2 + {\text{ade}}\underset{{{\text{k}}_{off} }}{\overset{{{\text{k}}_{on} }}{\rightleftharpoons}}IB*\cdot{\text{ade}}\underset{{{\text{k}}_{r} }}{\overset{{{\text{k}}_{f} }}{\rightleftharpoons}}B\cdot{\text{ade}}$$

This four-state model was further supported by RT SFX experiments that captured the structures of these states. The data analysis of the stopped-flow experiments provided detailed kinetic rate constants (Stagno et al. 2017): *k*_op_ = 2.1 s^−1^, *k*_cl_ = 0.53 s^−1^, *k*_on_ = 0.37 µM^−1^ s^−1^, *k*_off_ = 45 s^−1^, *k*_f_ = 132 s^−1^, *k*_r_ = 5.8 s^−1^, *sc* = 2.58 with the fitting error *Err(k,sc)* = 0.025, with *k*_*f*_ being the fastest rate in the four-state model, and the rest being on the subsecond-second timescale. Those rates are slower than conformation exchange rates, *k*_*ex*_, from relaxation measurements. Slower rates observed from the kinetic measurements may be attributed to an artifact caused by replacing U48 with 2-aminopurine (2AP) in the ligand-binding pocket. The fluorescent 2AP substation is used as a reporter to trace the trajectory of conformational changes triggered by ligand binding. Such a substitution in the binding pocket may perturb the kinetic landscape. Furthermore, the two methods might probe different types of motion on a different time scale. The much slower kinetic rates measured by the stopped-flow experiments might be relevant to such as domain-wise diffusive motion, whereas the CPMG measurements in this study probe micro- to milli-second timescale motion.

## Electronic supplementary material

Below is the link to the electronic supplementary material.

Supplementary material 1 (DOCX 1379 kb)

Supplementary material 2 (PPSX 7808 kb)

## Abbreviations

rA71: The 71-nucleotide aptamer domain of *add* adenine (A) riboswitch
CPMG: Carr–Purcell–Meiboom–Gill
XFEL: X-ray free electron laser
HSQC: Heteronuclear single quantum coherence
