# Supplementary material for: Conformational flexibility of adenine riboswitch aptamer in apo and bound states using NMR and an X-ray free electron laser
Source: J Biomol NMR. 2019 Oct 12;73(8):509–18. doi: 10.1007/s10858-019-00278-w (PMC6817744; doi:10.1007/s10858-019-00278-w)
Supplement: Supplementary file 1 — Supplementary material 1 (DOCX 1379 kb) [file 10858_2019_278_MOESM1_ESM.docx]

**Supporting Information**

**Conformational flexibility of adenine riboswitch aptamer in apo and bound states using NMR and an X-ray free electron laser**

**Jienv Ding^1*^, Monalisa Swain^1^, Ping Yu^1^, Jason R. Stagno^1^, Yun-Xing Wang^1*^**

*1. Protein-Nucleic Acid Interaction Section, Structural Biophysics Laboratory, Center for Cancer Research, National Cancer Institute, National Institute of Health, Frederick, Maryland 21702, USA*

* To whom correspondence should be addressed

Telephone: 3018465985; 3018467028 E-mail: [wangyunx@mail.nih.gov](mailto:wangyunx@mail.nih.gov); jienyu.ding@nih.gov

**Fig. S1** NMR assignments of ^2^J_NH_ ^1^H-^15^N HSQC spectra of rA71 in apo (**a**) and bound (**b**) states. The spectra were collected on Bruker 600 MHz with J coupling value of 25 Hz for INEPT transfer.


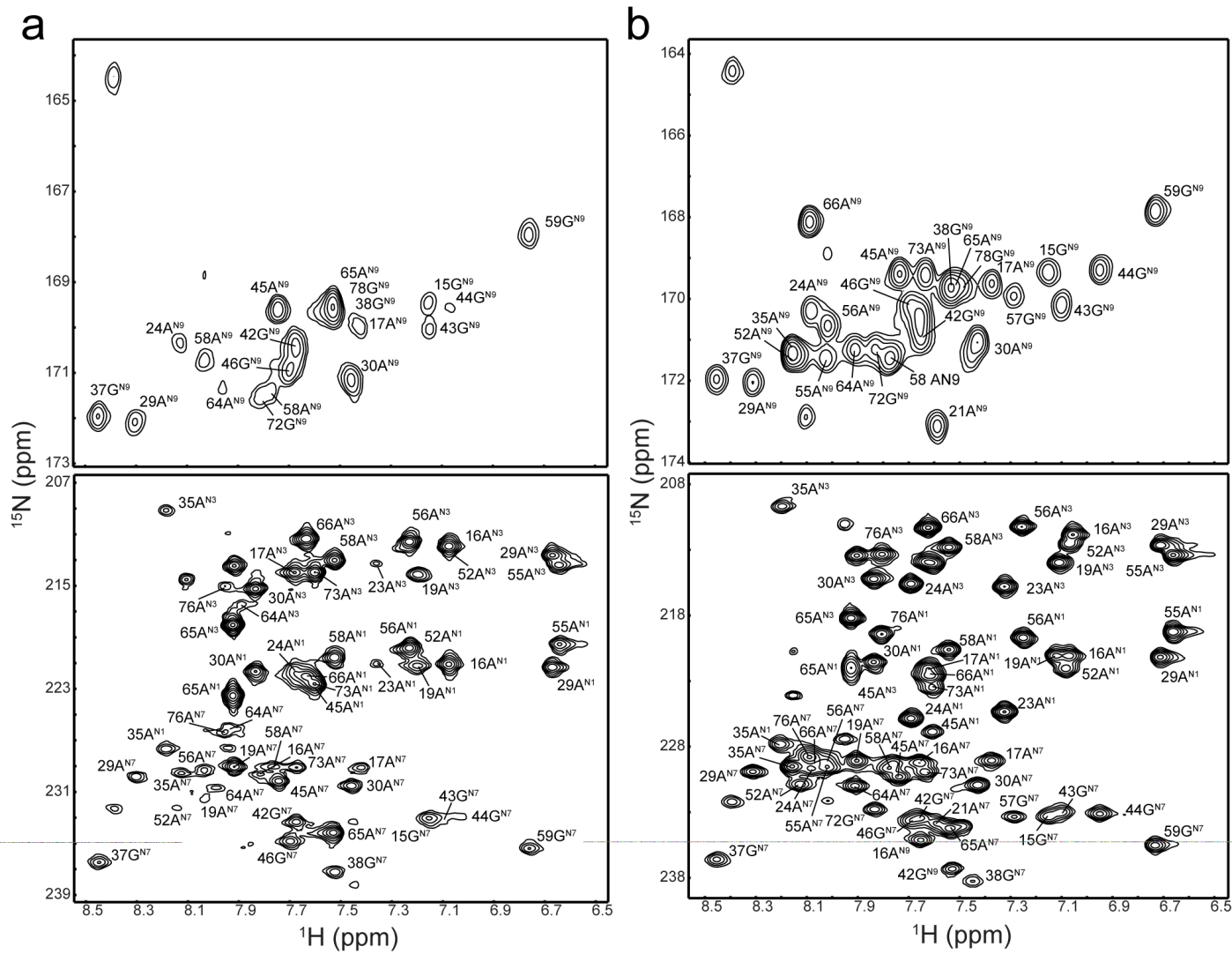


**Fig. S2** Plot of average B-factors per residue as reported in the crystal structures of rA71: apo1 (black)/apo2 (red) (room temperature; PDB 5E54), B•ade (blue) (room temperature; PDB 5SWE) and B•ade-cryo (green) (100 K; PDB 4TZX). The lower B-factors observed for B•ade-cryo illustrate how these parameters are dependent on temperature, thus highlighting the significance of using RT B-factors when compared with NMR data.

**
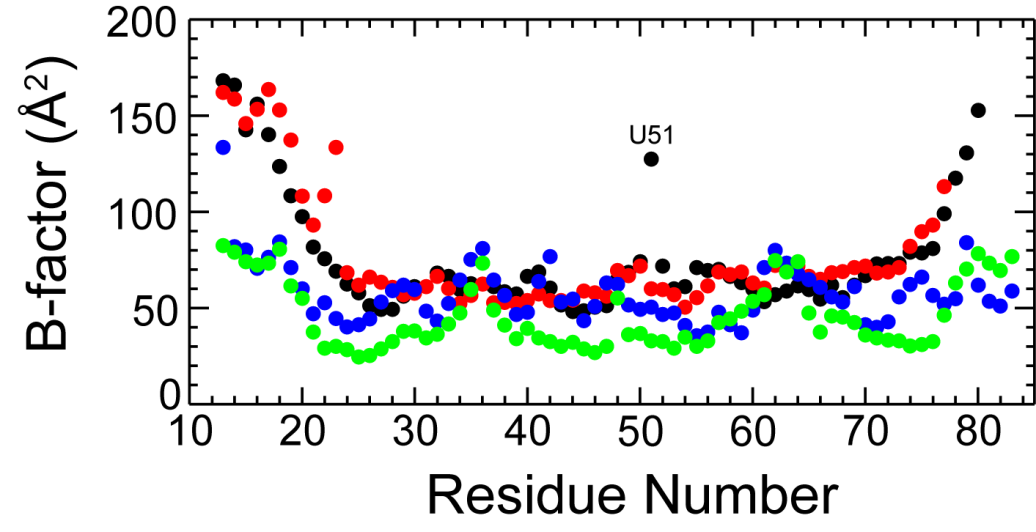
**

**Fig. S3** Global fitting curves for apo rA71. The imino ^15^N CPMG data were collected on Bruker 600 MHz (black) and 850 MHz (red).


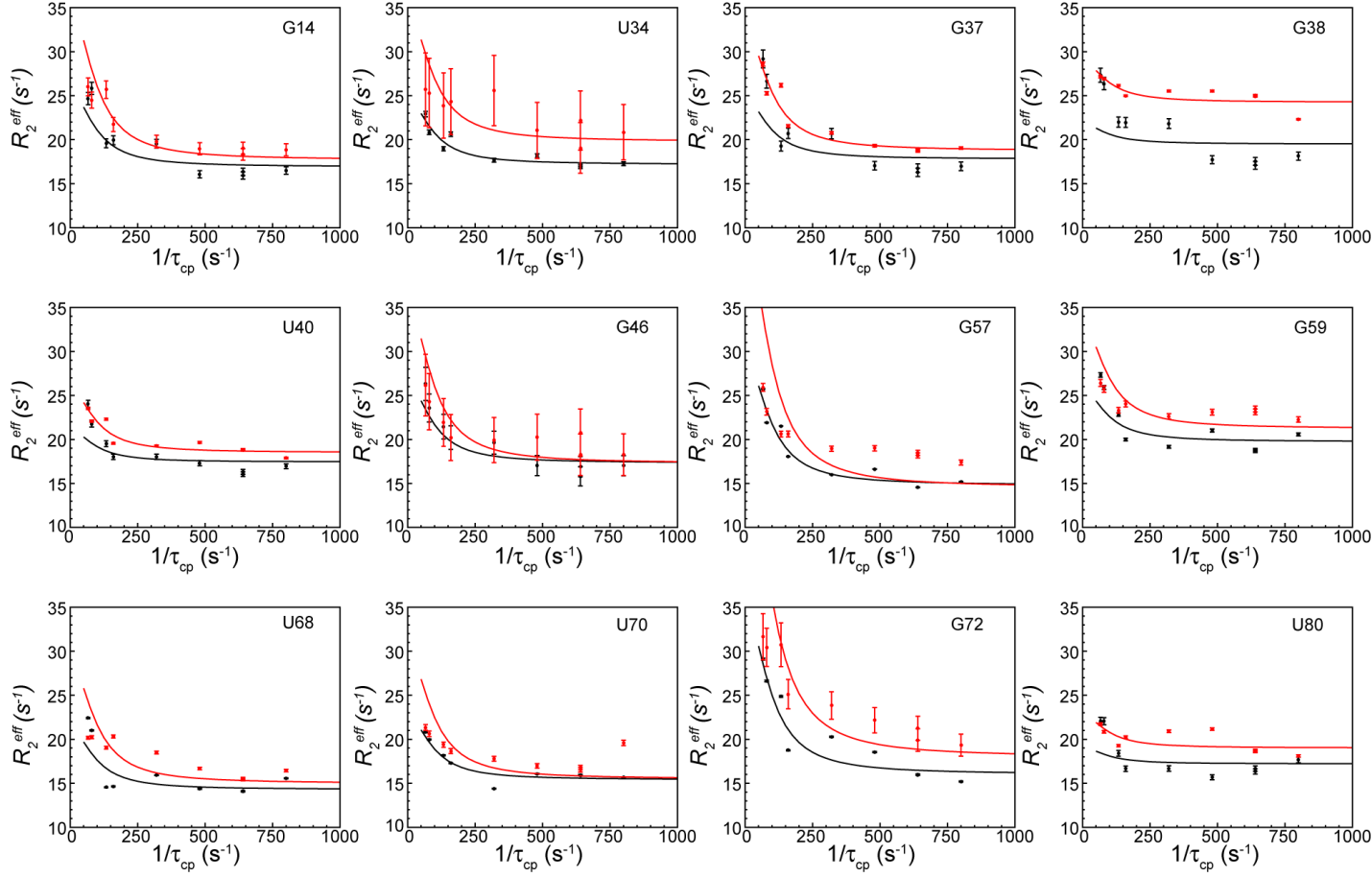


**Fig. S4** Sub-global fitting curves for holo rA71. The imino ^15^N CPMG data were collected on Bruker 600 MHz (black) and 850 MHz (red). Sub-global fittings are based on the segments shown in Table S2.


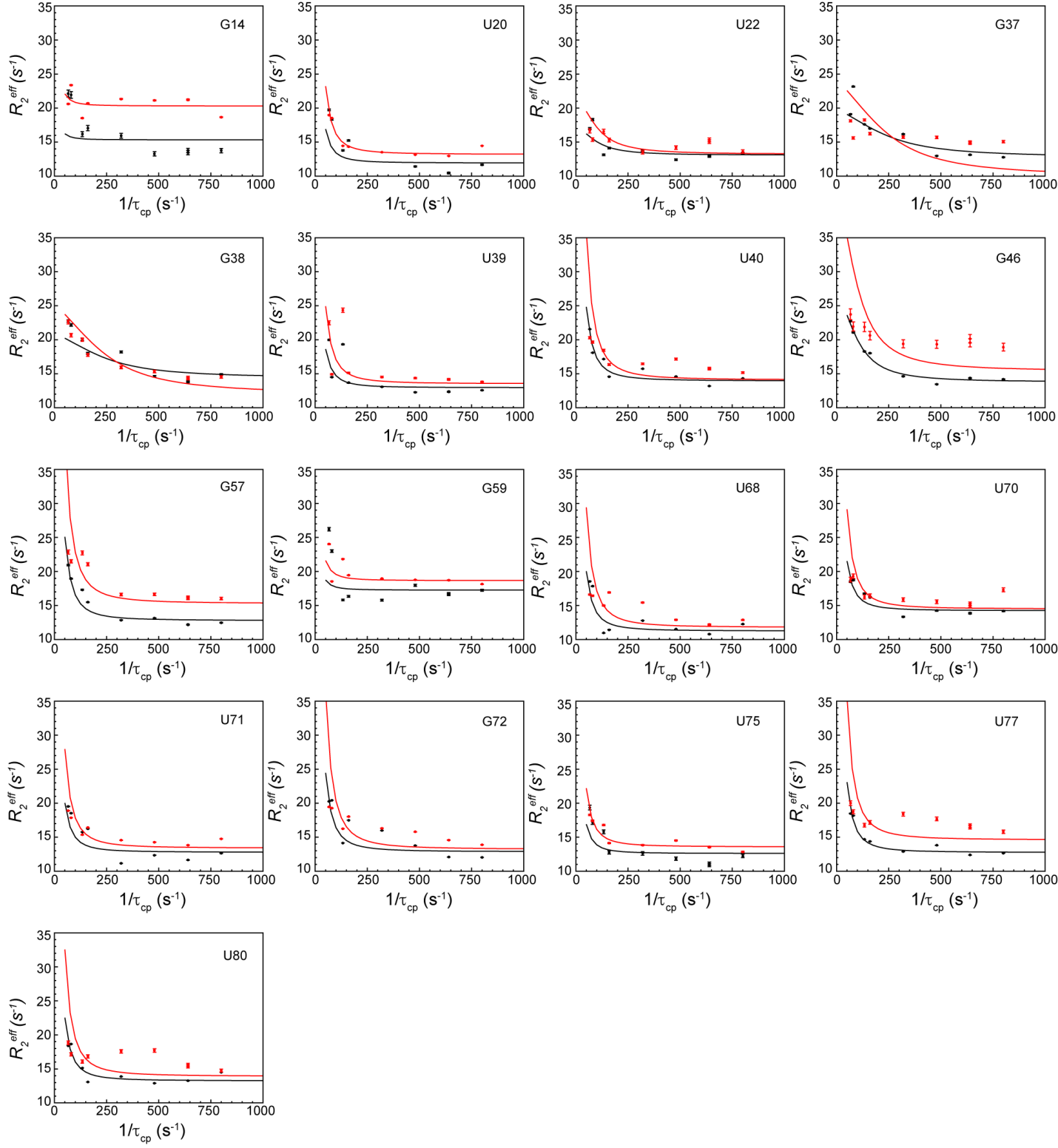


**Table S1** Individual fittings of rA71 in apo and bound states

| Apo | | Bound | |
| --- | --- | --- | --- |
|  | Individual fitting |  | Individual fitting |
| Residue | *k_ex_* (s^-1^) | Residue | *k_ex_* (s^-1^) |
| G14 | 448±72 | G14^*^ | 0.1±5.5×10^4^ |
| U34 | 363±51 | U20 | 79±1 |
| G37 | 407±22 | U22 | 139±31 |
| G38^*^ | 11982±4.3×10^7^ | G37 | 873±22 |
| U40 | 343±21 | G38 | 550±25 |
| U41^*^ | 12000±1.1×10^8^ | U39 | 487±18 |
| G46 | 325±1.6×10^2^ | U40 | 94±1 |
| G57 | 348±2 | G46 | 347±7 |
| G59 | 137±29 | G57 | 404±9 |
| U68 | 0.1±7.8×10^3^ | G59 | 1194±89 |
| U70 | 240±9 | U68 | 0.1±3.5×10^3^ |
| G72 | 318±6 | U70 | 332±16 |
| U79^*^ | 12000±8.3×10^7^ | U71 | 326±2 |
| U80^#^ | 11866±6.6×10^7^ | G72 | 74±1 |
|  |  | U75 | 270±14 |
|  |  | U77 | 199±2 |
|  |  | U80 | 81±0.4 |

Note:

^*^Residues not used for segmental or global fitting due to the poor data quality.

^#^U80 is at the end of the P1 helix that is highly dynamic in the Apo state.

**Table S2** Segmental/global *k_ex_* values and average B-factors for rA71 in apo and bound states

|  | Apo | | | Bound | | |
| --- | --- | --- | --- | --- | --- | --- |
|  | Residues | *k_ex_* (s^-1^) | B-factor(Å^2^) (apo1/apo2) | Residues | *k_ex_* (s^-1^) | B-factor(Å^2^) |
| P1 | 14,80 | 3184±32 | 123/131 | 20,75,77,80 | 83±0.03 | 69 |
| P2 | 40 | 343±21 | 56/58 | 39,40 | 94±0.14 | 54 |
| P3 | 57,59,68,70,72 | 318±1.5 | 66/66 | 57,59,68,70,71,72 | 89±0.05 | 45 |
| P2P3 |  | 318±1.5 | 61/62 |  | 89±0.05 | 50 |
| P1P3 |  | 318±1.5 | 98/98 |  | 84±0.02 | 59 |
| P1P2P3 |  | 318±1.5 | 84/84 |  | 84±0.02 | 58 |
| Loop | 34,37,38 | 430±11 | 62/62 | 37,38 | 804±4.9 | 65 |
| B.P | 46 | 325±156 | 71/74 | 22,46 | 335±5.4 | 52 |
| Loop+B.P |  | 429±11 | 66/68 |  | 491±3.4 | 59 |
| Global | all | 319±1.4 | 77/77 | all | 90±0.02 | 58 |

Note:

B.P: binding pocket

**Supplemental Movie**

The conformational flexibility of the 4 states of rA71, apo1 (blue), apo2 (cyan), IB•ade (yellow), and B•ade (magenta), based on time-averaged molecular dynamics ensemble refinement of the XFEL crystal structures using PHENIX^1^. Movies were generated using PyMOL^2^.

1. Adams, P. D. *et al.* PHENIX: building new software for automated crystallographic structure determination. *Acta Crystallogr. D* 58, 1948–1954 (2002).
2. The PyMOL Molecular Graphics System, Version 1.7.6.4 Schrödinger, LLC.
