# Supplementary figures and images for: Conformational flexibility of adenine riboswitch aptamer in apo and bound states using NMR and an X-ray free electron laser
Source: J Biomol NMR. 2019 Oct 12;73(8):509–18. doi: 10.1007/s10858-019-00278-w (PMC6817744; doi:10.1007/s10858-019-00278-w)

## Slide 1
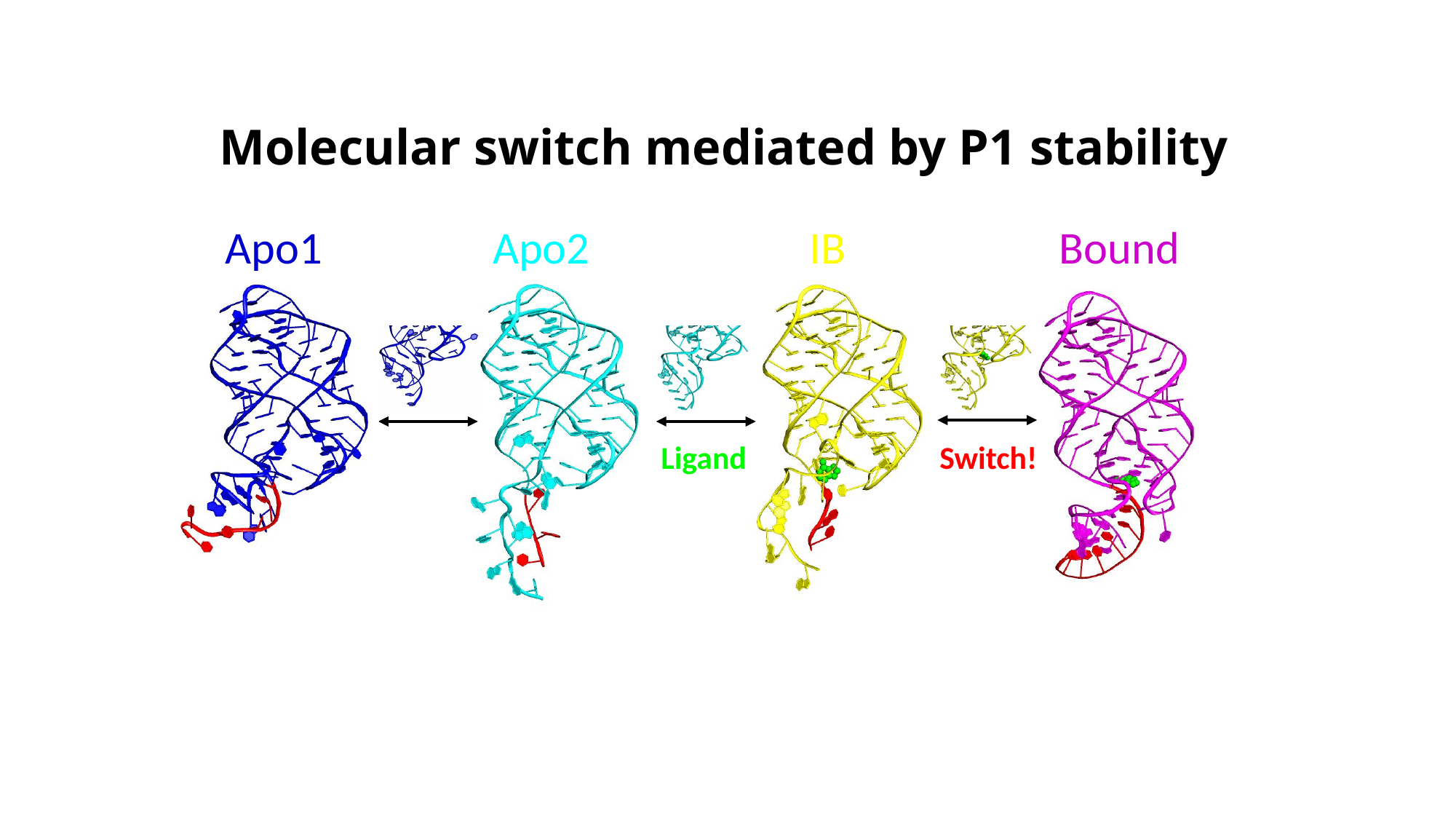

Supplement: Supplementary file 2 — Supplementary material 2 (PPSX 7808 kb) [file 10858_2019_278_MOESM2_ESM.ppsx]
